# Alpha-Lipoic Acid Inhibits Spontaneous Diabetes and Autoimmune Recurrence in Non-Obese Diabetic Mice by Enhancing Differentiation of Regulatory T Cells and Showed Potential for Use in Cell Therapies for the Treatment of Type 1 Diabetes

**DOI:** 10.3390/ijms23031169

**Published:** 2022-01-21

**Authors:** Shing-Hwa Huang, Shun-Li Kuo, Shyi-Jou Chen, Jeng-Rong Lin, Yuan-Wu Chen, Zhi-Jie Hong, Huey-Kang Sytwu, Gu-Jiun Lin

**Affiliations:** 1 Department of General Surgery, En Chu Kong Hospital, New Taipei 237, Taiwan; h610129@gmail.com; 2 Department of Biology and Anatomy, National Defense Medical Center, Taipei 114, Taiwan; 3 School of Traditional Chinese Medicine, Chang Gung University, Taoyuan 333, Taiwan; b9105001@cgmh.org.tw; 4 Center for Traditional Chinese Medicine, Chang Gung Memorial Hospital, Taoyuan 333, Taiwan; 5 Graduate Institute of Clinical Medical Sciences, College of Medicine, Chang Gung University, Taoyuan 333, Taiwan; 6 Graduate Institute of Life Sciences, National Defense Medical Center, Taipei 114, Taiwan; pedneuchen@hotmail.com (S.-J.C.); p8052@hotmail.com (J.-R.L.); 7 Department of Microbiology and Immunology, National Defense Medical Center, Taipei 114, Taiwan; sytwu@ndmctsgh.edu.tw; 8 Department of General Surgery, Tri-Service General Hospital, National Defense Medical Center, Taipei 114, Taiwan; lgf670822@office365.ndmctsgh.edu.tw; 9 National Defense Medical Center, School of Dentistry, Taipei 114, Taiwan; h6183@yahoo.com.tw; 10 Department of Oral and Maxillofacial Surgery, Tri-Service General Hospital, National Defense Medical Center, Taipei 114, Taiwan; 11 National Institute of Infectious Diseases and Vaccinology, National Health Research Institutes, Zhunan, Miaoli County 350, Taiwan

**Keywords:** α-lipoic acid, type 1 diabetes, non-obese diabetic mouse, regulatory T cells, islet transplantation, Treg-based cell therapy

## Abstract

Type 1 diabetes (T1D) is caused by the destruction of β cells in pancreatic islets by autoimmune T cells. Islet transplantation has been established as an effective treatment for T1D. However, the survival of islet grafts is often disrupted by recurrent autoimmunity. Alpha-lipoic acid (ALA) has been reported to have immunomodulatory effects and, therefore, may have therapeutic potential in the treatment of T1D. In this study, we investigated the therapeutic potential of ALA in autoimmunity inhibition. We treated non-obese diabetic (NOD) mice with spontaneous diabetes and islet-transplantation mice with ALA. The onset of diabetes was decreased and survival of the islet grafts was extended. The populations of Th1 cells decreased, and regulatory T cells (Tregs) increased in ALA-treated mice. The in vitro Treg differentiation was significantly increased by treatment with ALA. The adoptive transfer of ALA-differentiated Tregs into NOD recipients improved the outcome of the islet grafts. Our results showed that in vivo ALA treatment suppressed spontaneous diabetes and autoimmune recurrence in NOD mice by inhibiting the Th1 immune response and inducing the differentiation of Tregs. Our study also demonstrated the therapeutic potential of ALA in Treg-based cell therapies and islet transplantation used in the treatment of T1D.

## 1. Introduction

Autoimmune diabetes, formally referred to as type 1 diabetes (T1D), results from the destruction of insulin-producing β cells in the islets of the pancreas and has been identified as a T cell-mediated autoimmune disease [1]. The signs of T1D include hyperglycemia, high urine glucose polyuria, polydipsia, weight loss, abdominal symptoms, headaches and ketoacidosis. Chronic T1D also leads to many clinical complications including diabetic retinopathy, nephropathy, neuropathy, and macrovascular disease [2].The development of T1D is usually diagnosed in young patients; thus, this disease has also been termed “juvenile-onset” or “childhood-onset” diabetes. T1D only exhibits 30–50% concordance in monozygotic twins [3], thereby suggesting that both a genetic predisposition and environmental factors contribute to the pathogenesis of the disease [4,5,6]. NOD murine models have frequently been used for T1D studies. These mice have been bred to spontaneously develop T cell-dependent β cell destruction that resembles human T1D especially in the female NOD mice in which develop autoimmune diabetes around 80–90% at 40-weeks-old; therefore, female NOD mice often serve as animal models for studies concerning T1D [7].

The classical therapeutic strategy for patients with T1D is the administration of insulin injections to maintain normal levels of blood glucose. However, this approach is unable to provide real-time blood-glucose modulation and is ineffective for maintaining stable blood-glucose levels, which frequently leads to clinical complications [8]. Maintaining stable glucose levels is important to prevent the development of secondary complications that can result from T1D.

Islet transplantation has been reported as an effective strategy to achieve insulin independence, normoglycemia, and long-term homeostasis of blood glucose in T1D patients [9]. Moreover, islet transplantation is relatively simple to administer, as it does not require major surgical procedures. The procedure can be performed on an out-patient basis under local anesthesia and can be repeated several times without major discomfort to the patient [10]. Islet transplantation achieves nearly perfect blood-glucose monitoring and modulation in T1D patients [9]. However, the islet grafts are often destroyed by allogeneic graft rejection and autoimmune recurrence [11]. Autoreactive T cells harbor the memory of the β cells and are responsible for this autoimmune recurrence. Islet grafts in NOD mice have frequently experienced early graft failure because of the immediate destruction of the graft prior to immunological graft rejection [12,13]. Young et al. demonstrated that transplanted islet grafts in NOD mice were susceptible to recurrent autoimmunity as well [14]. Previous studies reported that human islets from genetically identical twins [15] or cadaver donors [16] had been destroyed by recurrent autoimmunity. Our previous study also showed that transplanted syngeneic NOD islet grafts were destructed by autoimmune recurrence in few days at NOD recipients [17]. Therefore, the development of a strategy to suppress autoimmune recurrence is critical for successful islet transplantation in patients with T1D.

Alpha-lipoic acid (ALA), also known as thioctic acid and 1,2 dithiolane-3-pentanoic acid, is a fatty acid containing rings; it is a naturally generated substance existing in almost all prokaryotic and eukaryotic cells and is also found in foods such as broccoli, spinach, and tomatoes [18,19]. It is a critical regulator of energy metabolism in mitochondria [20]. The physiologic function of ALA is as the co-factor of pyruvate dehydrogenase complex, which catalyzes the oxidative decarboxylation of α-keto acids such as pyruvate, α-ketoglutarate, or branched-chain α-keto acid [21]. Previous studies have demonstrated that ALA is also a potent antioxidant capable of scavenging reactive oxygen species (ROS) and chelating metal ions [22,23,24]. It is involved in the recycling of other cellular antioxidants including vitamins C and E as well as glutathione (GSH) [25]. These reports indicated that ALA exhibited a strong anti-oxidative effect. In addition to its anti-oxidative properties, it has been reported to elicit a modulatory effect on the immune system. ALA has demonstrated an anti-inflammatory effect in carrageenan-induced acute inflammation as well as in cotton pellet-induced chronic inflammation [26], and it has also been shown to attenuate LPS-induced monocyte activation and acute inflammatory responses [27]. Furthermore, other studies have suggested that ALA exhibited a modulatory effect in adoptive immunity. ALA lessens Th1-mediated inflammation in LPS-induced uveitis by reducing the release of Th1-related cytokines [28]. ALA prevented the development of experimental autoimmune encephalomyelitis (EAE) in a murine model of human multiple sclerosis (MS), though this effect was dose-dependent [29,30].Furthermore, in our previous study, ALA treatment at 50 mg/kg by intraperitoneal injection (i.p.) at day 1, 3, 5, and 7 ameliorated EAE by enhancing endogenous peroxisome-proliferator-activated receptor-γ [31]. ALA was also shown to inhibit T cell migration by suppressing the expression of ICAM-1 and VCAM-1 in endothelial cells in the central nervous system [32]. A recent study also demonstrated that ALA protected mice from concanavalin A-induced hepatitis by modulating the cytokine secretion and reducing ROS generation [33]. These reports indicated that ALA possesses anti-inflammatory and immunomodulatory effects, and these effects may be useful in the suppression of immune responses involved in autoimmunity as well as in allograft rejection.

Given these immunomodulation and anti-inflammatory effects, we further investigated whether ALA treatment could prevent the onset of spontaneous diabetes and prolong the survival of islet grafts in syngeneic and allogeneic islet-transplantation models.

## 2. Results

### 2.1. ALA Treatment Delayed the Onset of Autoimmune Diabetes

**Figure 1:**
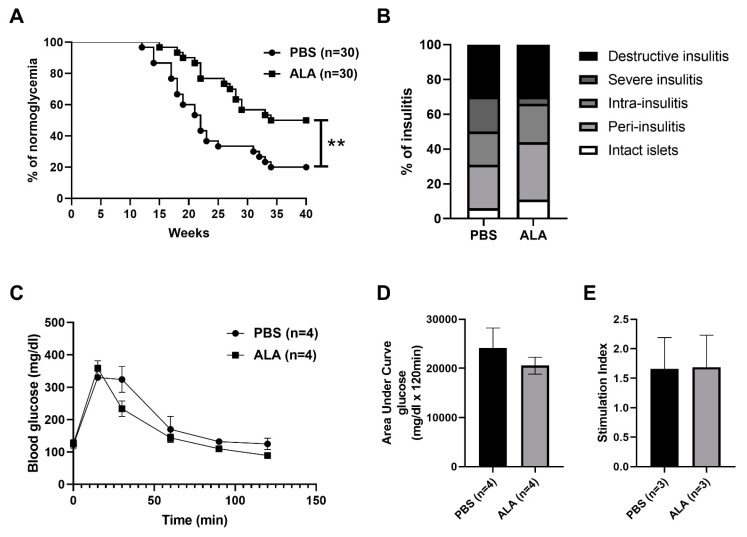
ALA treatment delayed spontaneous diabetic frequency in NOD mice. (**A**) Spontaneous diabetes in PBS-treat and ALA-treated NOD mice was monitored via weekly measurements of glycosuria. Diabetic frequency was significantly reduced in the ALA-treated group (*p* = 0.0033). (**B**) The severity of insulitis in PBS-treated or ALA-treated NOD mice was assessed by histological assay. The percentage of severe insulitis was lower in ALA-treated NOD mice, as compared to PBS-treated controls. (**C**) There were no significant differences in the metabolism of blood glucose between the PBS-treated and ALA-treated mice (*n* = 4; *p* = 0.8247 at 0 min, *p* = 0.2761 at 15 min, *p* = 0.0992 at 30 min, *p* = 0.5590 at 60 min, *p* = 0.0565 at 90 min, *p* = 0.1016 at 120 min) according to the IPGTT assay. (**D**) The area under the curve in the IPGTT assay also showed no significant differences between PBS-treated and ALA-treated groups. Data are expressed as means ± SEM (*n* = 4, *p* = 0.1603). (**E**) Islets isolated from PBS-treated and ALA-treated mice were stimulated with 2.8 mM or 16.7 mM glucose for 1 hr. The stimulation index was calculated by the ratio of insulin secreted at 16.7 and 2.8 mM glucose. The results showed no significant differences between PBS-treated and ALA-treated groups (*p* = 0.9714). Data are expressed as means ± SEM (*n* = 3, ** *p* < 0.01).

**Figure 2:**
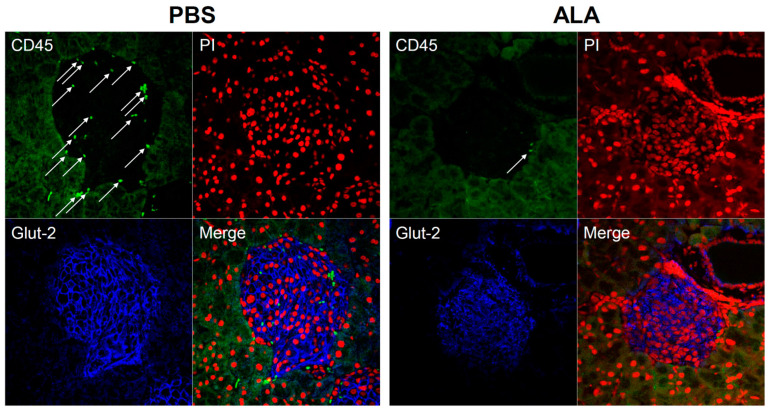
ALA treatment reduced the infiltration of leukocytes in the islet of NOD mice. Pancreas was harvested from 8-weeks-old NOD mice treated with PBS or ALA and embedded in paraffin. The pancreas-section slides were stained with antibodies against CD45 (Bright Green) and Glut-2 (Blue). The cell nucleus was counterstained with PI (Red). The slides were observed by a confocal microscope. White arrows indicated the presence of leukocytes (the signal of CD45). The infiltration of leukocytes was lesser in the islets of ALA-treated NOD mice when compared to PBS-treated mice (32 CD45 positive cells in PBS group vs. 2 CD45 positive cells in ALA group).

To evaluate the protective effect of ALA in autoimmune diabetes, thirty 4-weeks-old female NOD mice were treated with phosphate-buffered saline (PBS) or ALA (50 mg/kg) at two-day intervals for 4 weeks (from 4-weeks-old to 8-weeks-old). The incidence of spontaneous autoimmune diabetes was significantly lower, and the onset of disease was delayed in the ALA-treated group (Figure. 1A). Histological examination was performed to assess the effect of ALA treatment in the severity of insulitis. The percentage of severe insulitis was lower in ALA-treated NOD mice, as compared to the PBS-treated controls. In contrast, the percentage of intact islets was higher in the ALA-treated NOD mice (Figure 1B). The percentage of the insulitis degree in each stage and the number of counted islets are shown in Table 1. To confirm that the infiltration of leukocytes was decreased in the pancreatic islet of ALA-treated NOD mice, immunofluorescence assay (IFA) was performed to evaluate the infiltration of leukocytes. More leukocytes were infiltrated in the islet of PBS-treated NOD mice, as compared to the ALA-treated NOD mice (32 CD45 positive cells in PBS group vs. 2 CD45 positive cells in ALA group) (Figure 2). To evaluate whether ALA treatment affected the metabolism of blood glucose, an intraperitoneal glucose tolerance test (IPGTT) assay was performed for 6-weeks-old female NOD mice. There were no significant differences in the metabolism of blood glucose between the PBS-treated and ALA-treated mice according to the IPGTT assay (Figure 1C). The area under the curve (AUC) in the IPGTT assay also showed no significant difference between the PBS-treated and ALA-treated groups (Figure 1D). To investigate whether ALA treatment affected insulin production in the islets, we examined the insulin secretion in the islets that had been isolated from both the PBS-treated and ALA-treated mice. The stimulation index exhibited no significant differences between these two groups (Figure 1E). These results indicated that ALA treatment had effectively delayed the onset of autoimmune diabetes as well as the infiltration of leukocytes to the islets. Moreover, ALA treatment had not altered the production of insulin in the islets.

### 2.2. ALA Treatment Prolonged the Survival of Syngeneic and Allogeneic Islet Grafts after Islet Transplantation

**Figure 3:**
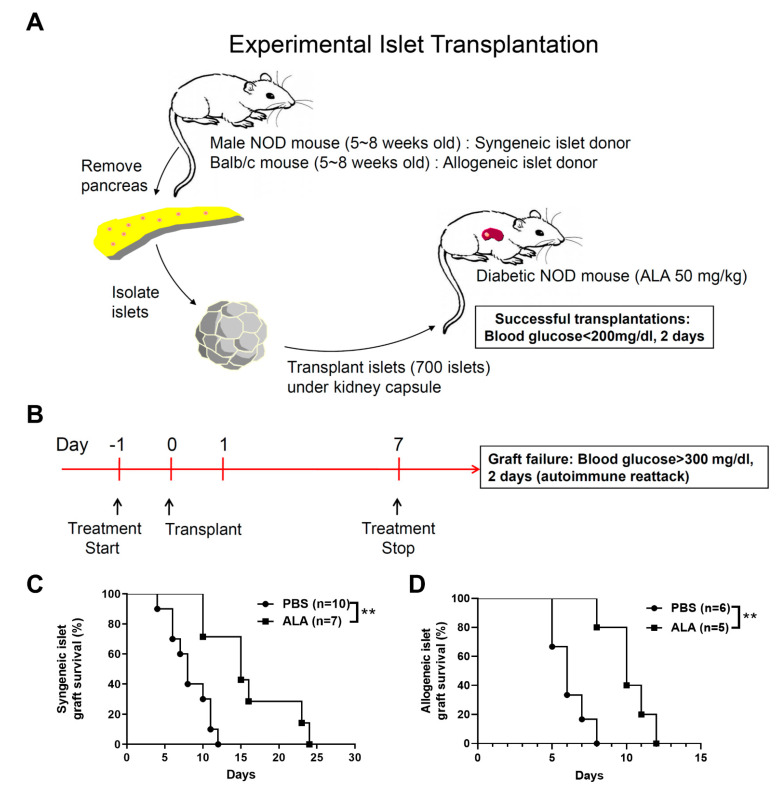
ALA treatment prolonged the survival of syngeneic and allogeneic islet grafts. (**A**) Isolated islets from male NOD or Balb/c mice were transplanted into the kidney subcapsular space of newly diabetic NOD recipients. (**B**) NOD recipients were also treated with ALA (50 mg/kg/day) once per day in syngeneic and allogeneic islet transplantations. The treatment was initiated at one day prior to islet transplantation (day −1). The entire treatment process was carried out until day 7 post islet transplantation (from day −1 to day 7) (**C**) The survival of syngeneic islet grafts was significantly extended in the ALA-treated NOD recipients (n = 10 in PBS vs. n = 7 in ALA, ** *p* = 0.0021). (**D**) The survival of allogeneic islet grafts was significantly extended in the ALA-treated NOD recipients (*n* = 6 in PBS vs. *n* = 5 in ALA, ** *p* = 0.0029).

To investigate whether ALA treatment protects β cells from autoimmune recurrence in islet transplantation, we performed syngeneic islet transplantation for diabetic NOD recipients. First, we isolated the islets from the male NOD mice (age: <8 weeks) and implanted these islets into the left kidney capsules of the newly diabetic female NOD recipients (Figure 3A). Next, the NOD recipients were treated with ALA (50 mg/kg/day) once per day. The treatment was initiated at one day prior to islet transplantation (day −1). The entire treatment process was carried out until day 7 post islet transplantation (from day −1 to day 7) (Figure 3B). The survival of the syngeneic islet graft was significantly extended in the ALA-treated recipients, as compared to the PBS-treated controls (Figure 3C). The mean graft survival time was 7.89 days in the PBS-treated recipients. In contrast, the mean graft survival time of the islet grafts in the ALA-treated recipients was 16.14 days (Table 2). To investigate whether ALA treatment also exhibited a protective effect in allogeneic islet transplantation, allogeneic islets were isolated from male Balb/c mice (age < 8 weeks) and then implanted into the left kidney capsules of newly diabetic female NOD recipients (Figure 3A). The NOD recipients were also treated with ALA (50 mg/kg/day) once per day. The treatment was initiated at one day prior to islet transplantation (day −1). The entire treatment process was carried out until day 7 post islet transplantation (from day −1 to day 7) (Figure 3B). The survival of the allogeneic islet grafts was significantly extended in the ALA-treated recipients, as compared to the PBS-treated controls (Figure 3D). The mean graft survival time was 6.17 days in the PBS-treated recipients. In contrast, the mean graft survival time of the islet grafts in the ALA-treated recipients was 10.2 days (Table 3). These data indicated that ALA treatment extended the survival of islet grafts in both syngeneic and allogeneic islet transplantations.

Since ALA is an effective anti-oxidant, we also examined whether ALA protects β cell from the damage of reactive oxidative species (ROS). We treated NIT-1 cells, a pancreatic β cell line derived from NOD mouse [34], with various concentrations of H_2_O_2_ for 24 h and measured the viability of NIT-1 cells by 3-(4,5-Dimethylthiazol-2-yl)-2,5-diphenyltetrazolium bromide assay (MTT assay). We found that 100 μM H_2_O_2_ treatment significantly induced the death of NIT-1 cells (Figure S1A). Next, we added 10 μM, 50 μM, and 100 μM of ALA into 100 μΜ H_2_O_2_-treated NIT-1 cells and measured the cell viability by MTT assay. Our results showed that ALA treatment did not provide a preventive effect in the ROS-induced cell death (Figure S1B).

### 2.3. ALA Treatment Reduced the Secretion of Inflammatory Cytokines and Increased the Production of Anti-Inflammatory Cytokines

**Figure 4:**
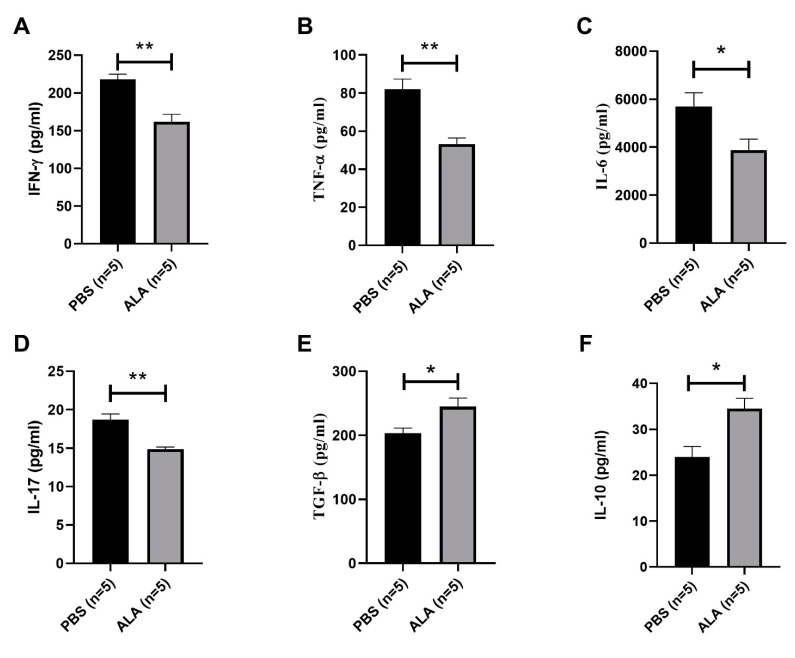
ALA treatment modulates cytokine secretion. Splenocytes were isolated from PBS-treated and ALA-treated NOD mice and stimulated with PMA and ionomycin. Culture medium containing secreted cytokines was measured by ELISA. The secretion of inflammatory cytokines (**A**) IFN-γ (** *p* = 0.0017), (**B**) TNF-α (** *p* = 0.0017), (**C**) IL-6 (* *p* = 0.0376) and (**D**) IL-17 was decreased in ALA-treated groups (** *p* = 0.0016). The secretion of anti-inflammatory cytokines (**E**) TGF-β (* *p* = 0.0313) and (**F**) IL-10 (* *p* = 0.0111) was increased in ALA-treated mice. Data are expressed as means ± SEM (*n* = 5; * *p* < 0.05, ** *p* < 0.01).

To investigate the immune modulatory effect of ALA in NOD mice, we examined the secretion of inflammatory and anti-inflammatory cytokines in the splenocytes of the PBS-treated and ALA-treated NOD mice. The secretion of inflammatory cytokines, such as interferon-γ (IFN-γ), tumor necrotic factor-α (TNF-α), interleukine-6 (IL-6) and interleukine-17 (IL-17) (Figure 4A–D) was reduced in the ALA-treated mice. In contrast, the production of anti-inflammatory cytokines interleukin-10 (IL-10) and transforming growth factor-β (TGF-β) was increased in ALA-treated NOD mice, as compared to PBS-treated controls (Figure 4E,F). These results indicated that ALA treatment reduced the production of inflammatory cytokines and increased the production of anti-inflammatory cytokines.

### 2.4. ALA Treatment Decreased the Population of Th1 Cells and Increased the Population of Tregs and IL-10-Producing CD4 T Cells

**Figure 5:**
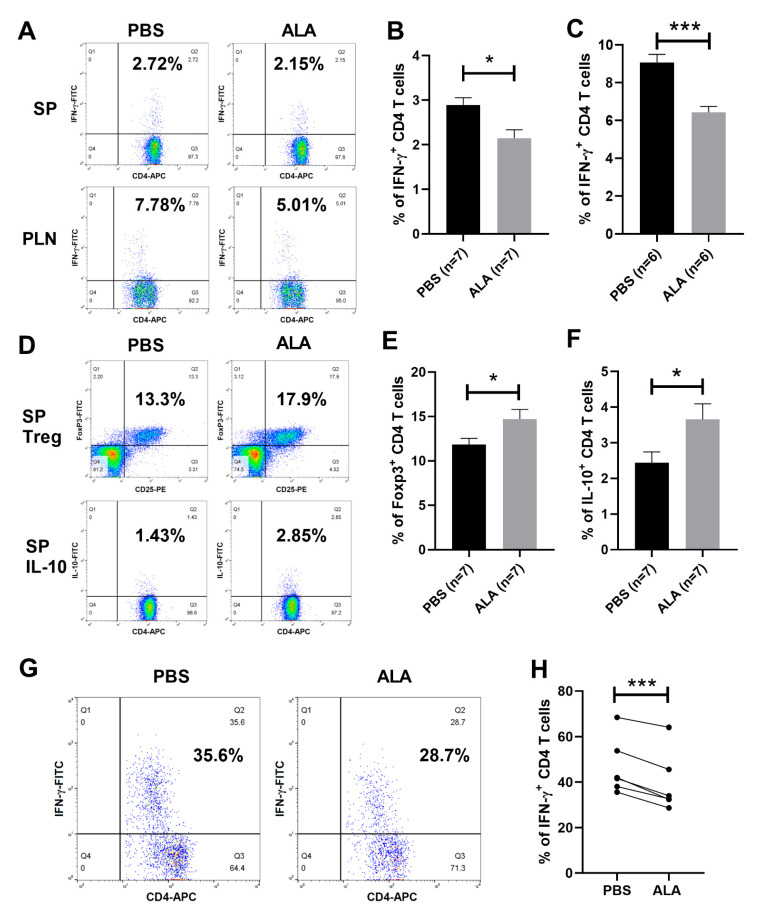
ALA treatment modulates the development of T cell subsets. NOD mice were treated with PBS or ALA. Lymphocytes isolated from spleens (SPs) of pancreatic lymph nodes (PLNs) of PBS-treated and ALA-treated NOD mice were stimulated with PMA plus ionomycin and monensin, and then fixed; permeabilized cells were stained with antibodies. The populations of T cell subsets were analyzed by flow cytometry. (**A**) Representative plots of the populations of Th1 cells in the spleens or PLNs of PBS-treated and ALA-treated NOD mice. (**B**) The percentage of splenic Th1 cells was significantly decreased in ALA-treated mice, as compared to the PBS-treated controls (*n* = 7, * *p* = 0.0111). (**C**) The percentage of Th1 cells in the PLNs was significantly decreased in ALA-treated mice, as compared to the PBS-treated controls (*n* = 6, *** *p* = 0.0005). (**D**) Representative plots of splenic Tregs and IL-10-producing CD4 T cells in PBS-treated and ALA-treated NOD mice. (**E**) The percentage of splenic Tregs was significantly increased in ALA-treated NOD mice, as compared to PBS-treated controls (*n* = 7, * *p* = 0.0445). (**F**) The percentage of IL-10-producing CD4 T cells in the spleen was significantly increased in ALA-treated NOD mice, as compared to the PBS-treated controls (*n* = 7, * *p* = 0.0418). (**G**) Representative plots of the populations of IFN-γ-producing CD4 T cells in the grafted sites of PBS-treated and ALA-treated NOD recipients. (**H**) The percentage of IFN-γ-producing CD4 T cells in the grafted sites of ALA-treated NOD recipients was significantly reduced, as compared to PBS-treated recipients (*n* = 6, *** *p* = 0.0003). Data are expressed as means ± SEM. (* *p* < 0.05, *** *p* < 0.001).

To further investigate the immune modulatory effect of ALA in the immune system of NOD mice, we examined the population of IFN-γ-producing CD4 T cells, as Th1 cells, in the spleen and pancreatic lymph nodes of the PBS-treated and ALA-treated NOD mice by flow cytometry (Figure 5A). The percentage of splenic and pancreatic lymph node Th1 cells was decreased in ALA-treated NOD mice, as compared to PBS-treated controls (Figure 5B,C). To investigate the effect of ALA in the regulatory T cell subsets, we analyzed the population of Tregs and IL-10-producing CD4 T cells in the spleens of NOD mice (Figure 5D). The percentages of both Tregs and IL-10-producing CD4 T cells were increased in the spleens of ALA-treated NOD mice, as compared to the PBS-treated controls (Figure 5E,F). We also investigated the modulatory effects of ALA in the Th1 cell populations of the NOD recipients. We analyzed the percentage of Th1 cells in the grafted sites of PBS-treated and ALA-treated NOD recipients by flow cytometry (Figure 5G). The percentage of Th1 cells in the grafted sites of ALA-treated NOD recipients was significantly decreased, as compared to the PBS-treated recipients (Figure 5H). These data indicated that in vivo ALA treatment encouraged the development of T cell subsets into regulatory T cells.

### 2.5. In Vitro ALA Treatment Induces the Differentiation of Tregs from the Naïve CD4 T Cells

**Figure 6:**
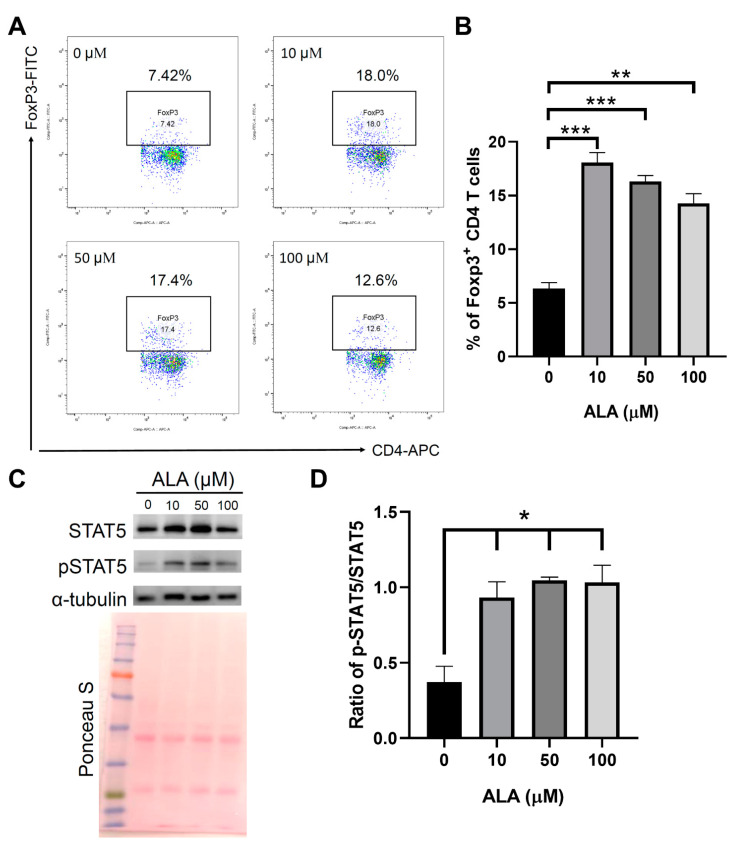
ALA treatment enhances in vitro Treg differentiation. Naïve CD4 T cells were isolated from female NOD mice and then cultured in anti-CD3 antibody coated (1 μg/mL) plate and combined with anti-CD28 antibody (1 μg/mL) plus human IL-2 cytokine (5 ng/mL) and TGF-β (5 ng/mL) with either PBS or different concentrations of ALA (10 μM, 50 μΜ, or 100 μM). (**A**) Representative plots of flow cytometry of in vitro Treg differentiation with or without ALA treatment. (**B**) The percentage of Tregs was significantly increased in the stimulation medium that contained ALA (n = 3; 0 μM vs. 10 μM *p* = 0.0004, vs. 50 μM *p* = 0.0002, vs. 100 μM *p* = 0.0017). (**C**) Representative figure of Western blot test of STAT5 and phosphorylated-STAT5 (p-STAT5). Alpha-tubulin was used as internal control. (**D**) The ratio between p-STAT5 and total STAT5 after normalization with α-tubulin. The ratio between p-STAT5 and total STAT5 was significantly increased in the stimulation medium that contained ALA (n = 3; 0 μM vs. 10 μM *p* = 0.0189, vs. 50 μM *p* = 0.0032, vs. 100 μM *p* = 0.0129). Data are expressed as means ± SEM. (* *p* < 0.05, ** *p* < 0.01, *** *p* < 0.001).

To investigate whether in vitro ALA treatment promotes the differentiation of Tregs from the naïve CD4 T cells, we isolated naïve CD4 T cells from the female NOD splenocytes and incubated these cells in an anti-CD3 antibody-coated plate and induced Treg differentiation by adding IL-2 and TGF-β with either PBS or different concentrations of ALA (10, 50, or 100 μM). We found that ALA treatment enhanced the differentiation efficiency of the Tregs (Figure 6A,B). To investigate whether the promotion of Treg differentiation by ALA was a result of enhanced phosphorylation of signal transducer and activator of transcription 5 (STAT5), Western blotting was performed to evaluated the status of the STAT5 phosphorylation of STAT5 in the in vitro Treg differentiation. Our results indicated that ALA treatment enhanced the phosphorylation of STAT5 in the process of Treg differentiation (Figure 6C,D).

### 2.6. Adoptive Transfer of ALA-Enhanced In Vitro Differentiated Tregs Exhibited a Better Protective Effect Than the Cells without ALA Treatment

**Figure 7:**
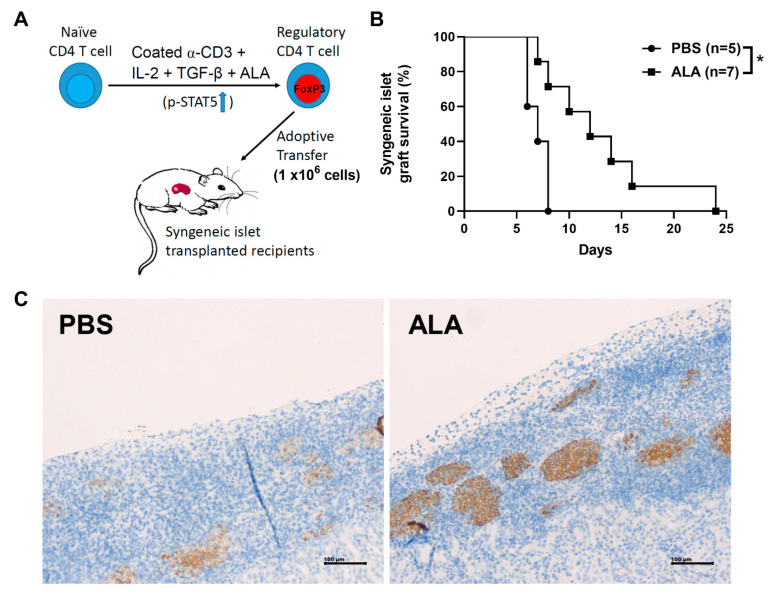
The adoptive transfer of ALA-enhanced differentiated Tregs and its impact on the islet graft survival in syngeneic islet transplantation. (**A**) Naïve CD4 T cells were isolated from the splenocytes of female NOD mice. The naïve CD4 T cells were cultured under stimulation condition with PBS or 50 μΜ of ALA. A total of 1 × 10^6^ PBS-treated and ALA-treated cells were collected and adoptively transferred into the NOD mice at day 1 and day 3 post-islet transplantation. (**B**) The islet graft survival was significantly extended in the ALA-treated group (*n* = 7, * *p* < 0.05), as compared to the PBS-treated group (*n* = 5, * *p* = 0.0107). (**C**) The secretion of insulin was assessed by immunohistochemical staining. Red arrow indicates the insulin staining in the PBS-treated group and the ALA-treated Treg-transferred NOD recipients.

Naïve CD4 T cells were isolated from the splenocytes of the female NOD mice. The naïve CD4 T cells were cultured under in vitro Treg differentiation conditions with either PBS or 50 μΜ of ALA. We adoptively transferred 1 × 10^6^ differentiated Tregs at day 1 and day 3 post-islet transplantation (Figure 7A). Islet survival was significantly extended in the ALA-treated cell-transferred groups, as compared to the PBS-treated cell-transferred group (Figure 7B). Immunohistochemical assays showed more insulin-producing islets in the ALA-treated cell-transferred recipients (Figure 7C). The individual islet graft survival time is presented in Table 4. These results indicated that the adoptive transfer of ALA-enhanced differentiated Tregs into NOD recipients exhibited a protective effect for the islet grafts. Our data showed that the adoptive transfer of in vitro ALA-enhanced differentiated Tregs exhibited a protective benefit, indicating its potential in Treg-based cell therapy in islet transplantation for type 1 diabetes patients.

## 3. Discussion

T1D is an autoimmune disease caused by the destruction of islet β cells by autoreactive T cells [1]. However, most T1D patients are diagnosed after diabetes onset. Islet transplantation has been used as an effective therapy for T1D [10] due to its lower cost as well as being a safer option, as compared to the pancreatic transplantation [9,35]. Recurrent autoimmunity plays a leading role in islet-graft destruction and impairs the survival of islet grafts in human islet transplantation. Moreover, the destruction of grafts by autoimmune recurrence often takes place earlier than that of allogeneic graft rejection [11,36]. Therefore, it is important to overcome recurrent autoimmunity for both syngeneic and allogeneic islet transplantation.

ALA is an organic compound produced in plants, animals, and humans in nature. It acts in the Krebs cycle, plays important roles in various chemical reactions, and serves as a cofactor of some enzymatic complexes that involved in energy generation for the cell [37]. ALA was shown to be beneficial against mild-to-moderate diabetic sensorimotor polyneuropathy [38]. The studies in animal models have shown that ALA also exhibits immune modulatory effects [39]. In this study, we investigated the preventive effect of ALA treatment in the onset of spontaneous diabetes and the inhibitory role of the autoimmune recurrence in islet graft rejection. To our knowledge, this may be the first study demonstrating showing that ALA treatment effectively suppressed the onset of autoimmune diabetes and extended the survival of islet grafts. Our results also showed that ALA treatment reduced the population of Th1 cells in the spleen and pancreatic lymph node of NOD mice. Furthermore, this treatment increased the percentages of Tregs and IL-10-producing CD4 T (T regulatory 1, Tr1) cells in the splenocytes, thereby suggesting that ALA promoted increased generation of Tregs and Tr1 cells. The increase in the population of Tregs may explain the suppressive effect of ALA on the population of Th1 cells as well as in the production of IFN-γ.

In addition, we found that ALA treatment enhanced the differentiation of Tregs from naïve CD4 T cells, thereby demonstrating an enhancing effect of ALA in the production of Tregs. Therefore, we further investigated the underlying mechanisms involved in the enhancement of Treg differentiation by ALA. A previous study reported that the STAT5 signaling pathway may be required for the development of Tregs [40], and another study also demonstrated that STAT5 polarization promoted Treg generation [41]. These results highlight the importance of the STAT5 signaling pathway in the production of Tregs. Therefore, we examined whether ALA-promoted Treg differentiation was a result of enhanced activation of the STAT5 signaling pathway. The levels of STAT5 protein and phosphorylated STAT5 had indeed increased following ALA treatment, which indicated that ALA may promote differentiation of Tregs via enhanced activation of the STAT5 signaling pathway.

The adoptive transfer of in vitro differentiated Tregs has been used to treat autoimmune or inflammatory diseases in other animal models. In systemic lupus erythematosus (SLE) murine models, the adoptive transfer of ex vivo-expanded Tregs delayed the onset of renal complications and extended survival [42,43]. Tregs have effectively inhibited both the proliferation of myelin oligodendrocyte glycoprotein (MOG)-specific Th1 cells and their production of cytokines, and the adoptive transfer of Tregs conferred significant protection from the impact of EAE [44]. The expansion of myelin-reactive Tregs prevented disease relapse when it occurred after the onset of clinical EAE [45]. Our previous study also demonstrated that the adoptive transfer of Tregs suppressed the formation of encapsulated peritoneal sclerosis in a murine model [46]. The results of this study demonstrated that in vitro ALA treatment enhanced the differentiation of Tregs and the adoptive transfer of these Tregs extended the survival of islet grafts in the NOD recipients.

In clinical settings, Treg-based cell therapies are currently undergoing clinical trials for the treatment of autoimmune diseases and transplant rejection [47]. Phase I and II clinical trials of polyclonal Treg therapy in T1D and other autoimmune diseases have shown that this therapy can be safe and effective [48]. A phase I clinical trial of polyclonal Treg infusion for six individuals showed no infusion reactions or high-grade cell-therapy–related adverse events [49], which suggested that Treg-based cell therapy could be safely administered in humans. Another clinical trial involving children with T1D showed that the adoptive transfer of polyclonal, autologous Tregs extended the survival of pancreatic islets for one year [50]. One of the major challenges for this therapy has been how to more effectively induce the differentiation of Tregs from naïve CD4 T cells or peripheral leukocytes in vitro [47]. The results of our study demonstrated that ALA does enhance the in vitro differentiation of Tregs from splenocytes and that these cells exhibited a protective effect for islet grafts in islet transplantation. Our data provided the preclinical evidence for the application of ALA in Treg-based cell therapy for T1D in islet transplantation.

## 4. Materials and Methods

### 4.1. Animal Model

The NOD/ShiLtJ strain, commonly called NOD mice, is a polygenic model for autoimmune type 1 diabetes. It was purchased from Jackson Laboratory (Bar Harbor, ME, USA) and subsequently bred at the animal center of the National Defense Medical Center in Taipei, Taiwan, under specific pathogen-free conditions. Balb/c mice were purchased from the National Laboratory Animal Center in Taipei, Taiwan.

### 4.2. Preparation of ALA Solution and In Vivo Treatment of ALA

ALA in powder form was obtained from Sigma (Sigma-Aldrich, St Louis, MO, USA), stored at room temperature and dissolved in 1 N sodium hydroxide before use. This solution was titrated with 2 M HCl to a pH of 7.2 and an approximate salt concentration of 0.9% (*w*/*v*). All solutions of ALA were sterile filtered. The mice were administered ALA that was freshly diluted in sterile phosphate-buffered saline (PBS) (Sigma-Aldrich) (1 mg/100 µL/mouse/i.p. injection, equivalent to 50 mg/kg/mouse/i.p. injection). The treatment dosage was based on our previous study in the treatment of EAE [31]. The control mice were injected with an equal volume of PBS at the same time points.

### 4.3. Islet Isolation and Transplantation

Diabetic NOD females with blood-glucose concentrations 300–500 mg/dL for two consecutive days were selected as recipients, and NOD male mice aged 5–8 weeks were used as islet donors. Islets were purified from 6-week-old male NOD mice (it is easier to acquire sufficient number of intact islets) using the collagenase-digesting method as described previously in [51]. Collagenase buffer was prepared with Hank’s balanced salt solution containing 1.5 mg/mL collagenase (Sigma-Aldrich) and injected into the pancreas via a common bile duct. The pancreas was digested in a 37 °C water bath for 20 min, and then the islets were separated according to a density gradient using a Histopaque 1077-1 (Sigma-Aldrich). Islets with a diameter between 75 μm and 250 μm were handpicked using a dissecting microscope. Finally, we collected a total of approximately 650 islets that were then implanted into the left renal capsules of newly diabetic NOD female mice with blood glucose concentrations of 300–500 mg/dL. For allogenic transplantation, islets were purified from 6-week-old male Balb/c mice and transplanted into the left renal capsules of newly diabetic NOD female mice.

### 4.4. Urine Glucose and Blood Glucose Monitoring and Assessment of Insulitis

Urine glucose concentrations (glycosuria) were measured weekly using Chemstrips (Boehringer Mannheim, Indianapolis, IN, USA). Mice with urine glucose concentration >27.75 mmol/L on two consecutive tests were defined as diabetic. Blood glucose concentrations were monitored daily by Roche ACCU-CHEK (Roche Ltd., Basel, Switzerland) after islet transplantation. Graft rejection and loss of function were defined as blood glucose levels higher than 300 mg/dL for two consecutive days. For the assessment of insulitis, pancreatic tissues were obtained from 14-week-old PBS-treated or ALA-treated NOD mice and the severity of insulitis was scored on haematoxylin–eosin stained sections (Sigma-Aldrich) and classified as described [52]. The degree of insulitis in the pancreas was evaluated by scoring 15–30 islets/mouse in a blinded fashion according the following criteria: intact islet: no mononuclear cell infiltration; peri-insulitis: mononuclear cell infiltration in <25%; intra-insulitis: mononuclear cell infiltration in 25–50% of the islet; severe insulitis: mononuclear cell infiltration in 50–75% of the islet; destructive insulitis: >75% of the islet was infiltrated.

### 4.5. Naïve CD4 T Cell Sorting

Naïve CD4 T cells were harvested and sorted from the spleen of the NOD mice by magnetic cell-separation beads. The BD IMagTM Mouse CD4 T Lymphocyte Enrichment Set-DM (BD Biosciences, San Jose, CA, USA) was used for the negative selection of the CD4 T lymphocyte by removing non-CD4 T cells from splenocytes. Cells were then re-suspended at a concentration of 1 × 10^6^ cells/mL in RPMI1640 medium (Gibco, Amarillo, TX, USA) supplemented with 10% fetal bovine serum and 1% penicillin (Sigma-Aldrich) and streptomycin (Sigma-Aldrich). For the isolation of naïve CD4 T cells, the selected CD4 T cells were placed in biotinylated mouse CD4 T lymphocyte antibody (BD Biosciences) and 2 μL biotinylated anti-CD25 antibody (BD Biosciences) and then incubated for 15 min. After being washed with the medium, 5 μL streptavidin particles were added into the tube for 30 min and refrigerated at 4 °C. The cells were then placed on the magnetic cell-separation platform for 8 min; this process was repeated three times. The positive fractions of the cells were isolated as naïve CD4 T cell.

### 4.6. In Vitro Treg Differentiation

Naïve CD4 T cells were harvested and sorted from the splenocytes of female NOD mice and then cultured for 3 days in anti-CD3 antibody-coated (1 μg/mL) (BD Biosciences) plate, to which anti-CD28 antibody (1 μg/mL) (BD Biosciences) plus human IL-2 cytokine (5 ng/mL) (PeproTech, Inc., East Windsor, NJ, USA) and TGF-β (5 ng/mL) (PeproTech, Inc.) with either PBS or different concentrations of ALA (10 μM, 50 μM, and 100 μM) were added. CD4^+^CD25^+^Foxp3^+^ cells were then measured from these PBS- or ALA-treated naïve CD4^+^ T cells by flow cytometry.

### 4.7. Flow Cytometry

Lymphocytes were harvested from spleen, pancreatic lymph nodes or islet grafts. For Foxp3 staining, 1 × 10^6^ cells were first stained with 2 μg/mL allophycocyanin (APC)-conjugated anti-mouse CD4 (clone GK1.5) (eBioscience Inc., San Diego, CA, USA), phycoerythrin (PE)-conjugated anti-mouse CD25 (clone PC61) (eBioscience Inc.) in 100 μL of flow buffer for 30 min at 4 °C, and then fixed and permeabilized overnight with 1 mL Fixation/Permeabilization working solution (eBioscience Inc.). After fixation and permeabilization, the cells were stained with 5 μg/mL fluorescein isothiocyanate (FITC)-conjugated anti-Foxp3 (clone FJK-16S) (eBioscience Inc.) in 100 μL permeabilization buffer. For intracellular cytokine staining, the cells were stimulated for 4–6 h with 20 ng/mL phorbol 12-myristate 13-acetate (PMA) (Sigma-Aldrich), 1 μM ionomycin (Sigma-Aldrich), and 4 μM monensin (BD Biosciences). The 1 × 10^6^ stimulated cells were stained with 2 μg/mL antibody to surface CD4-APC in 100 μL of flow buffer on ice for 25–30 min (in the dark), and washed with 1 mL of FACS buffer (PBS containing 0.5% FBS). After being washed, the cells were fixed overnight with 0.2 mL of IC Fixation Buffer (eBioscience Inc.). The cells were then stained with 5 μg/mL anti-mouse IL-10-PE, IFN-γ –FITC-conjugated antibodies (eBioscience Inc.) on ice for 30 min in 100 μL permeabilization buffer. Flow cytometric analysis was performed with a FACS Calibur (BD Pharmingen, Franklin Lakes, Bergen, NJ, USA) and FlowJo software 8.7.1 (Treestar, Ashland, OR, USA).

### 4.8. ELISA Detection of Cytokines

Splenocytes were isolated from PBS-treated and ALA-treated NOD mice, separately, and stimulated with PMA and ionomycin for 6 hrs. Culture medium containing secreted cytokines was measured by ELISA kit (eBioscience Inc.) according to the manufacturer’s instructions.

### 4.9. Adoptive Transfer of Regulatory T Cells

The 1 × 10^6^ differentiated Tregs were adoptively transferred into the diabetic NOD mice after islet transplantation by intraperitoneal (i.p.) injection. This protocol was followed according to our previous studies [46,53].

### 4.10. Protein Extraction and Western Blot

The protein samples were extracted from the splenocytes of the NOD mice that were incubated in anti-CD3 antibody-coated (1 μg/mL) (BD Biosciences) plate and added with anti-CD28 antibody (1 μg/mL) (BD Biosciences) plus human IL-2 cytokine (5 ng/mL) and TGF-β (5 ng/mL) with either PBS or different concentrations of ALA (10 μM, 50 μM or 100 μΜ) by using the PROPREP™ Protein Extraction Solution (iNtRON Biotechnology, Gyeonggido, Korea). A sample in the protein extraction solution was homogenized by an Ultrasonic Homogenizer (Misonix, Farmingdale, NY, USA), and it was then incubated on ice for 20–30 min to lyse the cells. After centrifugation at 13,000 rpm for 10 min at 4 °C, the supernatant was transferred to a new Eppendorf tube. A total of 10 μg of the protein sample was separated on 10% sodium dodecyl sulfate polyacrylamide gel electrophoresis (SDS-PAGE), and then transferred to a polyvinylidene difluoride (PVDF) membrane (Millipore, Billerica, MA, USA). The membrane was blocked with 5% skim milk at room temperature for 1 h and was then incubated in a buffer with a rabbit anti-STAT5 antibody (GeneTex Inc., Alton Pkwy Irvine, CA, USA), anti-acetylated H3 (GeneTex Inc.) or a mouse anti-β-actin antibody (GeneTex Inc.) overnight. After washing with PBST (0.05% Tween20 in PBS) three times, the membrane was incubated in the hybridization buffer with a horseradish peroxidase (HRP)-conjugated goat anti-rabbit IgG antibody (1:2000; Santa Cruz Biotechnology, Inc., Dallas, TX, USA) or a HRP-conjugated goat anti-mouse IgG antibody (Santa Cruz Biotechnology, Inc.) for 1 h. The membrane was subsequently washed with PBST three times. After incubation with the chemiluminescent HRP substrate (Millipore), the signals were detected by the LAS-3000 imaging system (Fujifilm, Tokyo, Japan).

### 4.11. Immunofluorescence Assay

Pancreas was harvested from 8-weeks-old NOD mice treated with PBS or ALA and embedded in paraffin. Primary antibodies against CD45 (a common marker for leukocytes) and Glucose transporter 2 (Glut-2, a marker of islet β cells) were used to observe the presence of leukocytes in the islet of pancreas. The pancreas-section slides were stained with Alexa fluor 488-conjugated anti-CD45 antibody (eBioscience Inc.) and Alex fluor 350-conjugated anti-Glut-2 antibody (eBioscience Inc.) over night at 4 °C. The cell nucleus was counterstained with propidium iodide (PI) (eBioscience Inc.). Finally, the slides were analyzed by a confocal microscopy.

### 4.12. Immunohistochemical Assays

Kidneys transplanted with islets were harvested from NOD recipients and then embedded in paraffin (Sigma-Aldrich). The kidney-section slides were stained with antibodies against insulin (abcam, Cambridge, USA, ab7842) overnight and then stained with a second anti-guinea pig IgG (Bethyl, A60-110p) antibodies (abcam, Cambridge) for 1 h. Finally, the slides were stained with hematoxylin (Sigma-Aldrich) and analyzed via light microscopy.

### 4.13. Statistical Analysis

GraphPad Prism 8 (GraphPad Software, San Diego, CA, USA) was used as statistical software in this study. The data are presented as the mean ± standard error of mean (SEM). The significance of islet graft survival time between the PBS-treated and ALA-treated groups was determined via Kaplan–Meier survival analysis. The significance of diabetic frequency between the PBS-treated and ALA-treated NOD mice was also determined via Kaplan–Meier survival analysis. For the remaining experiments, the *p*-values were calculated using a two-tailed Student’s t-test. Differences were considered significant at *p* < 0.05.

## 5. Conclusions

Our results demonstrated that ALA treatment had significantly prevented the onset of diabetes and prolonged islet graft survival in NOD mice. To understand the mechanisms involved in this protective effect, we then investigated the influence of ALA on immune cells in regard to cytokine profiles and the populations of T cells. ALA treatment reduced the proportion of T helper 1 (Th1) cells and increased the proportion of regulatory T cells (Tregs) in the spleens of NOD mice. We further demonstrated that ALA increased the differentiation of Tregs from the naïve CD4 T cells of NOD mice. Overall, ALA treatment reduced diabetic incidence in NOD mice and prolonged the survival of syngeneic and allogeneic islet grafts. This protective effect was associated with increased differentiation of Tregs in the modulation of immune cell effector functions. Furthermore, the ability of ALA to induce Treg differentiation could be beneficial in the Treg-based cell therapy for islet-transplantation treatment in type 1 diabetes.

In conclusion, our study demonstrated, possibly for the first time, that ALA inhibited the onset of spontaneous diabetes and the autoimmune recurrence seen in islet transplantation that is often used in the treatment of T1D. ALA had an immune modulatory effect by suppressing the Th1 immune response and inducing Treg differentiation through the enhanced activation of the STAT5 signaling pathway. Furthermore, the adoptive transfer of in vitro ALA-induced Tregs exhibited a similar therapeutic effect, as compared to the in vivo ALA treatment, demonstrating the therapeutic potential of ALA in Treg-based cell therapies and islet transplantation used in the treatment of T1D.

## Figures and Tables

**Table 1:** The percentages of the degree of insulitis in each stage.

Stage of Insulitis	% of PBS	Number	% of ALA	Number
Intact islet	6%	5	11%	11
Peri-insulitis	25%	23	33%	33
Intra-insulitis	19%	17	22%	22
Severe insulitis	20%	18	4%	4
Destructive insulitis	30%	27	30%	30

**Table 2:** The survival time of syngeneic islet graft in the NOD recipients.

Group	Individual Graft Survival Time (Days)	Number	Average Survival Time
PBS	4, 6, 6, 7, 8, 8, 10, 11, 11, 12	10	7.89
ALA	10, 10, 15, 15, 16, 23, 24	7	16.14

**Table 3:** The survival time of allogeneic islet graft in the NOD recipients.

Group	Individual Graft Survival Time (Days)	Number	Average Survival Time
PBS	5, 5, 6, 6, 7, 8	6	6.17
ALA	8, 10, 10, 11, 12	5	10.2

**Table 4:** The survival time of islet grafts in PBS- or ALA-treated cell transferred NOD recipients.

Group	Individual Graft Survival Time (Days)	Number	Average Survival Time
PBS	6, 6, 7, 8, 8	5	7
ALA	7, 8, 10, 12, 14, 16, 24	7	13

## Data Availability

All data underlying this article are available directly in the article text.

## Supplementary Materials

The following are available online at https://www.mdpi.com/article/10.3390/ijms23031169/s1.
