# Supplementary material for: Alpha-Lipoic Acid Inhibits Spontaneous Diabetes and Autoimmune Recurrence in Non-Obese Diabetic Mice by Enhancing Differentiation of Regulatory T Cells and Showed Potential for Use in Cell Therapies for the Treatment of Type 1 Diabetes
Source: Int J Mol Sci. 2022 Jan 21;23(3):1169. doi: 10.3390/ijms23031169 (PMC8835933; doi:10.3390/ijms23031169)
Supplement: Supplementary file 1 [file ijms-23-01169-s001.zip › ijms-1514675-Supplementary material.pdf]

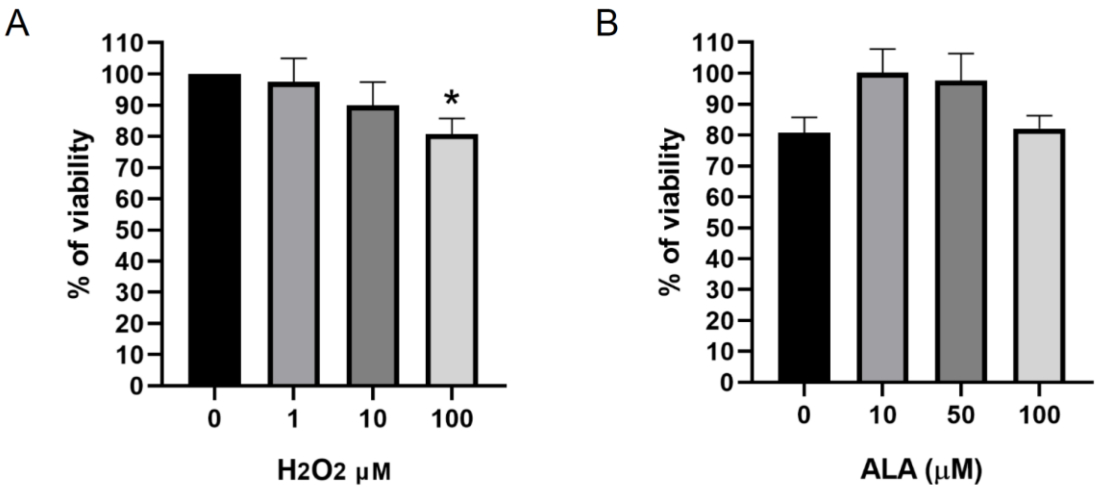

Figure S1. ALA treatment did not improve the survival of ROS-induced cell death in NIT-1 cell line. (**A**) NIT-1 cells were treated with various concentrations of H_2O_2 for 24 hours and measured the viability of NIT-1 cells by 3-(4,5-Dimethylthiazol-2-yl)-2,5-diphenyltetrazolium bromide assay (MTT assay). We found that 100 µM H_2O_2 treatment significantly induced the death of NIT-1 cells (n = 3; 0 µM vs. 1 µM p = 0.7536, vs. 10 µM p = 0.2516, vs. 100 µM * p = 0.0187). (**B**) Different concentrations of ALA (10 µM, 50 µM, and 100 µM) was added into 100µM H_2O_2 -treated NIT-1 cells and measured the cell viability by MTT assy. Our results showed that ALA treatment did not provide a preventive effect in the ROS-induced cell death (n = 3; 0 µM vs. 10 µM p = 0.0970, vs. 50 µM p = 0.1702, vs. 100 µM p = 0.8433).

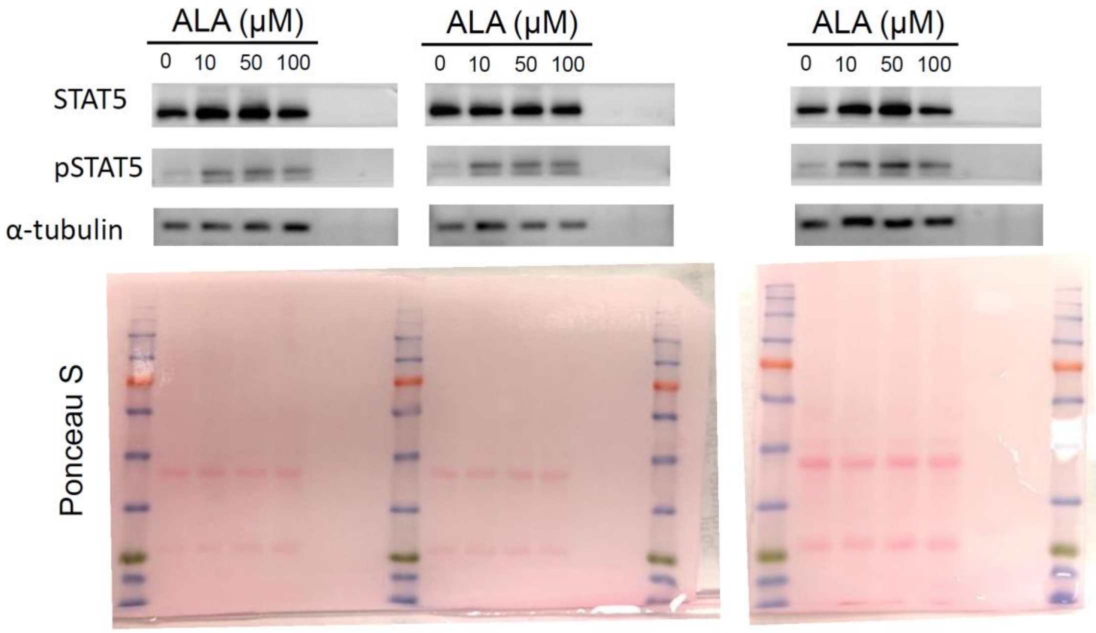

Figure S2. Three independent experiments of Western blotting for phosphorylated STAT5.

Negative in urine glucose with normoglycemia

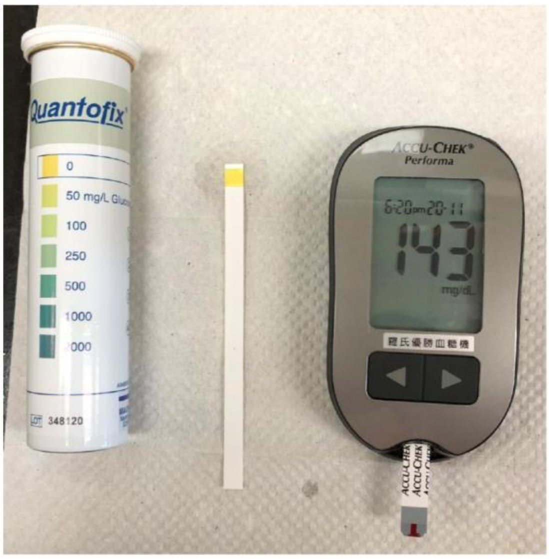

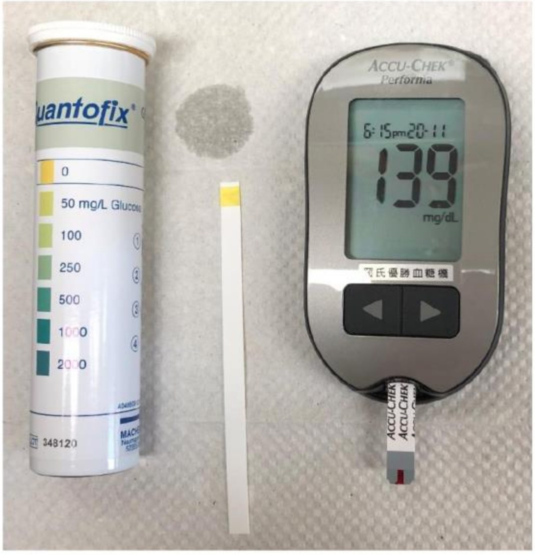

0–50 mg/L urine glucose with 353 mg/dL blood glucose

1000 mg/L urine glucose with 454 mg/dL blood glucose

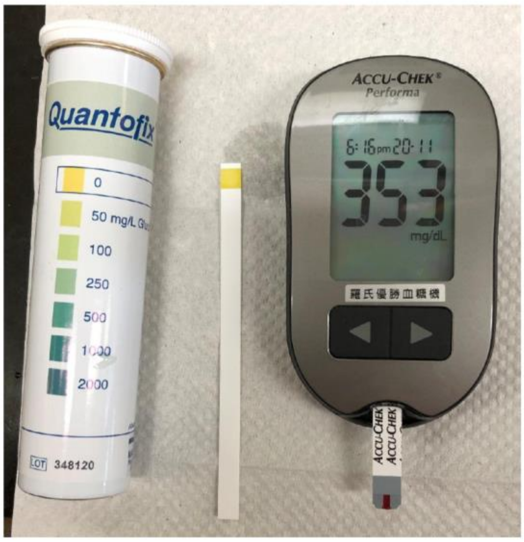

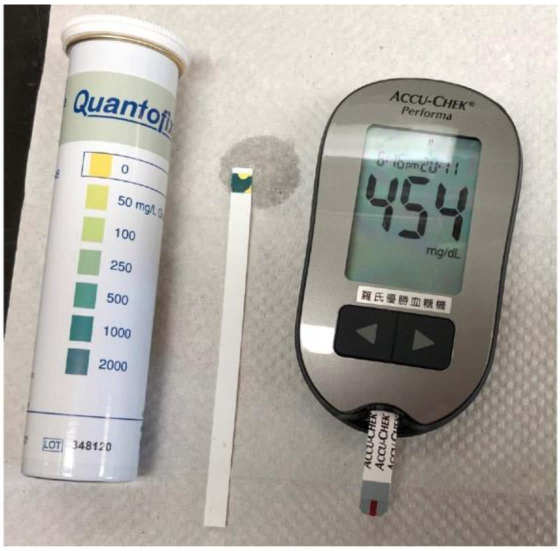

500–1000 mg/L urine glucose with 443 mg/dL blood glucose

500–1000 mg/L urine glucose with 455 mg/dL blood glucose

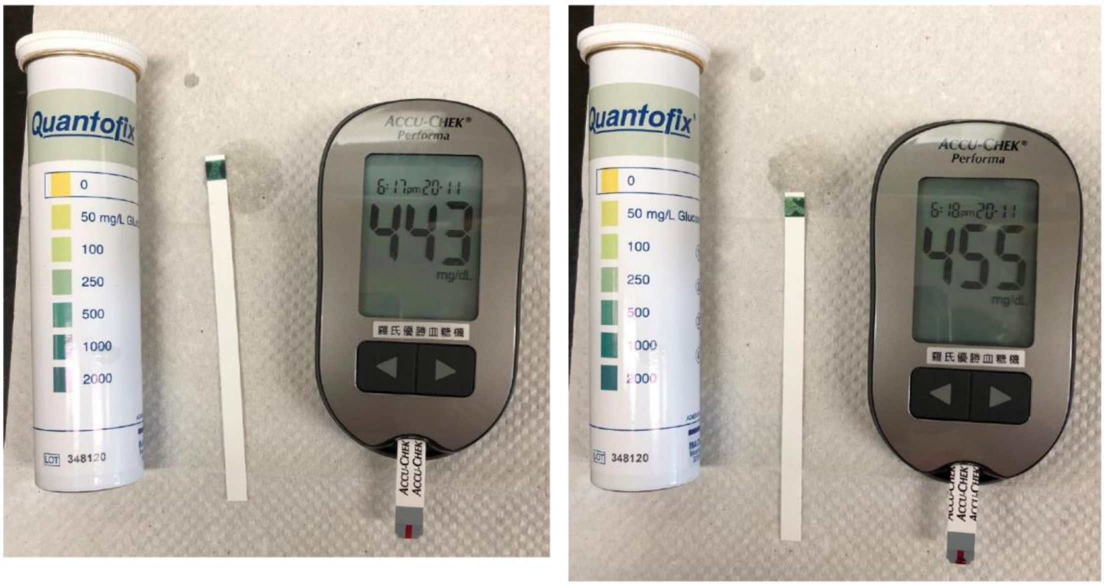

2000 mg/L urine glucose with blood glucose higher than 600 mg/dL

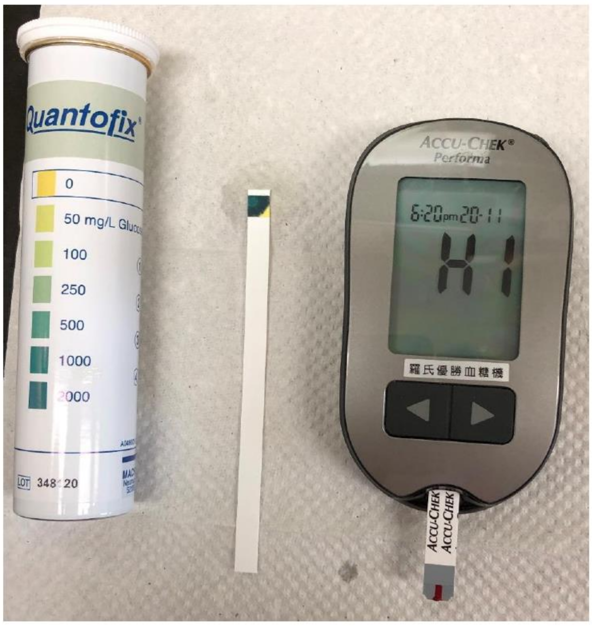

Figure S3. The correction between urine glucose and blood glucose in NOD mice.
